# Cytotoxic effect and molecular docking of 4-ethoxycarbonylmethyl-1-(piperidin-4-ylcarbonyl)-thiosemicarbazide—a novel topoisomerase II inhibitor

**DOI:** 10.1007/s00894-012-1679-6

**Published:** 2012-11-28

**Authors:** Agata Siwek, Paweł Stączek, Monika Wujec, Krzysztof Bielawski, Anna Bielawska, Piotr Paneth

**Affiliations:** 1 Department of Organic Chemistry, Faculty of Pharmacy, Medical University, Chodźki 4a, 20-093 Lublin, Poland; 2 Department of Genetics of Microorganisms, University of Lodz, Banacha 12/16, 90-237 Lodz, Poland; 3 Department of Medicinal Chemistry and Drug Technology, Medical University of Białystok, Kilinskiego 1, 15-089 Białystok, Poland; 4 Institute of Applied Radiation Chemistry, Faculty of Chemistry, Lodz University of Technology, Zeromskiego 116, 90-924 Lodz, Poland

**Keywords:** Thiosemicarbazide derivative, Human topoisomerase II, Cytotoxicity, Molecular docking, DFT calculation

## Abstract

The preliminary cytotoxic effect of 4-ethoxycarbonylmethyl-1-(piperidin-4-ylcarbonyl)-thiosemicarbazide hydrochloride (**1**)—a potent topoisomerase II inhibitor—was measured using a MTT assay. It was found that the compound decreased the number of viable cells in both estrogen receptor-positive MCF-7 and estrogen receptor-negative MDA-MB-231breast cancer cells, with IC_50_ values of 146 ± 2 and 132 ± 2 μM, respectively. To clarify the molecular basis of the inhibitory action of **1**, molecular docking studies were carried out. The results suggest that **1** targets the ATP binding pocket.

**Electronic supplementary material:**

The online version of this article (doi:10.1007/s00894-012-1679-6) contains supplementary material, which is available to authorized users.

**Figure:**
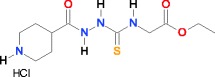
4-ethoxycarbonylmethyl-1-(piperidin-4-ylcarbonyl)-thiosemicarbazide hydrochloride

## Introduction

An important chemotherapeutic target in the treatment of cancer is the ATP-dependent enzyme topoisomerase II (Topo II), which regulates the conformational changes in DNA topology necessary for transcription, replication, and chromosome condensation and segregation [1, 2]. Currently, six Topo II inhibitors (etoposide, teniposide, doxorubicin, daunorubicin, idarubicin, and mitoxantrone) are prescribed as highly anti-neoplastic drugs in clinical use. However, emerging tumor resistance and several side effects, such as hematological toxicity, nausea and vomiting, and hair loss, are associated with them [3, 4]. Therefore, efforts have been made by many research groups to find new chemicals with improved bioactivity. Although many compounds were found as cytostatic agents and Topo II and(or) I inhibitors, most showed significant toxicity for normal dividing cells, which precludes their use as drugs or lead molecule candidates [5]. The discovery of new types of Topo II inhibitors that can be synthesized easily, show increased sensitivity in drug resistant tumors and decreased dose-limiting toxicities would be a significant addition to the choices available in the treatment of cancer.

Recently, as part of a search for the molecular basis of antibacterial activity of thiosemicarbazide-based compounds, we documented for the first time that one of the mechanisms of the bioactivity of these molecules is connected with inhibition of bacterial Topo IV [6–8]. Since both bacterial and human topoisomerases share a unique structural ATP-binding motif, called the Bergerat fold [9], we decided to conduct enzymatic studies to determine the effect of thiosemicarbazide derivatives on human topoisomerases. We found that the title compound, 4-ethoxycarbonylmethyl-1-(piperidin-4-ylcarbonyl)-thiosemicarbazide hydrochloride (**1**) selectively inhibited Topo II activity almost completely at 15 μM concentration, proving more potent than etoposide under the same experimental conditions [10]. To expand our initial findings with further details on the biological action pathways of **1**, the compound was further assessed by preliminary cytotoxicity measurements. Herein we present the results of these investigations, as well as those of subsequent docking studies and DFT calculations, which allowed us to suggest that compound **1** targets the ATP binding pocket.

## Materials and methods

Etoposide and camptothecin were purchased from TopoGEN (http://www.topogen.com/). Stock cultures of breast cancer MCF-7 and MDA-MB-231 were purchased from the American Type Culture Collection (Rockville, MD). Dulbecco’s minimal essential medium (DMEM) and foetal bovine serum (FBS) used in cell culture were products of Gibco (http://www.invitrogen.com/site/us/en/home/brands/Gibco.html). Glutamine, penicillin and streptomycin were obtained from Quality Biologicals (Gaithersburg, MD). [^3^H]-Thymidine (6.7 Ci/mmol) was the product of NEN (Boston, MA).

### Cell culture

Human breast cancer MDA-MB-231 and MCF-7 cells maintained in DMEM supplemented with 10 % fetal bovine serum (FBS), 50 U/ml penicillin, 50 μg/ml streptomycin at 37 °C. Cells were cultured in Costar flasks and subconfluent cells were detached with 0.05 % trypsin and 0.02 % EDTA in calcium-free phosphate buffered saline, counted in hemocytometers and plated at 5 × 10^5^ cells per well in six-well plates (Nunc, http://www.nuncbrand.com) in 2 ml of growth medium (DMEM without phenol red with 10 % CPSR1). Cells reached about 80 % of confluency at day 3 and in most cases such cells were used for the assays.

### Cytotoxic assay

To examine the effect of the studied drugs on MCF-7 and MDA-MB-231cell proliferation, the cells were seeded in 24-well tissue culture dishes at 1 × 10^5^ cells/well with 1 ml growth medium. After 48 h (1.8 ± 0.1 × 10^5^ cells/well) plates were incubated with varying concentrations of compound **1**, chlorambucil and 0.5 μCi [^3^H]-thymidine for 24 h at 37 °C. Cells were rinsed three times with PBS, solubilised with 1 ml 0.1 M sodium hydroxide containing 1 % SDS, scintillation fluid (9 ml) was added and radioactivity incorporation into DNA was measured in a scintillation counter.

### Cell viability assay

The assay was performed according to the method of Carmichael [11] using 3-(4,5-dimethylthiazole-2-yl)-2,5-diphenyltetrazolium bromide (MTT) (Sigma, St. Louis, MO). Confluent cells, cultured for 24 h with various concentrations of the studied compounds in six-well plates were washed three times with PBS and then incubated for 4 h in 1 ml MTT solution (5 mg/ml PBS) at 37 °C in 5 % CO_2_ in an incubator. The medium was removed and 1 ml 0.1 M HCl in absolute isopropanol was added to attached cells. Absorbance of converted dye in living cells was measured at a wavelength of 570 nm. Cell viability of breast cancer cells cultured in the presence of ligands was calculated as a per cent of control cells.

### Automated docking setup

Docking was performed by means of the FlexX program [12] as implemented in LeadIT software package [13] using models of Topo II binding site complexed with AMP-PNP (PDB id 1ZXN [14]) and etoposide (PDB id 3QX3 [15]) as templates. The ligand within the active site and all water molecules were removed while magnesium ion was allowed to remain with the charge of +2. The active site was defined to include all atoms within 6.5 Å radius of the native ligand. The first 100 top ranked docking poses were saved for each docking run. Subsequently, compounds **1**–**3** were docked using same docking parameters. Both rigid and flexible docking was performed (the results for the latter are presented). For compounds **1** and **3**, both piperidine protonated and deprotonated forms were considered.

### DFT calculations

Following the methodology used previously [16] energies of best poses of compounds **1**–**4** obtained from FlexX docking were calculated using the B3LYP functional [17, 18] expressed in the basis set of 6–31 G(d) [19–21] as implemented in Gaussian 09 [22] to describe their molecular properties. Natural population analysis (NPA) phase of NBO was used [23]. The highest occupied (HOMO) and lowest unoccupied (LUMO) molecular orbitals were illustrated using the GaussView 5.0 program [24].

### Statistical analysis

In all experiments, the mean values for six independent experiments ± standard deviations (SD) were calculated, unless otherwise indicated. The results were submitted to statistical analysis using Students t-test, accepting *P* < 0.05 as significant.

## Results and discussion

**Fig. 1:**
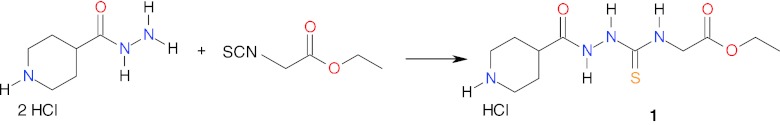
Synthetic route for 4-ethoxycarbonylmethyl-1-(piperidin-4-ylcarbonyl)-thiosemicarbazide hydrochloride (**1**)

The titled 4-ethoxycarbonylmethyl-1-(piperidin-4-ylcarbonyl)-thiosemicarbazide hydrochloride, **1**, was synthesized through the route outlined in Fig. 1; details of the synthetic procedure, structural characterization of **1** and its effect on human topoisomerase II activity are described in our patent application [10].

### Cytotoxic effect of 1

**Fig. 2:**
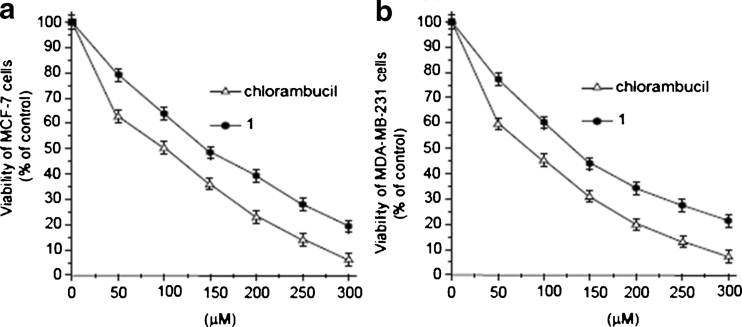
Viability of MCF-7 (**a**) and MDA-MB-231 (**b**) cells treated for 24 h with different concentrations of compound **1** and chlorambucil. Mean values ± SD from three independent experiments performed in duplicate are presented

Many reports in recent years have indicated that inhibitory action towards Topo II coincides with cytotoxic properties for several naturally occurring and synthetic compounds [25–30]. We therefore subjected **1** to a preliminary MTT assay in estrogen receptor-positive (MCF-7) and estrogen receptor-negative (MDA-MB-231) breast cancer cells to identify any possible correlation. Compound **1** decreased the number of viable cells in both MCF-7 and MDA-MB-231 breast cancer cells; however, at somewhat higher concentrations than the positive control chlorambucil. The inhibitory activities (IC_50_) of compound **1** on MDA-MB-231 and MCF-7 were 146 ± 2 and 132 ± 2 μM, respectively, whereas that of chlorambucil were 92 ± 2 and 97 ± 2 μM, respectively, as shown in Fig. 2. Although the cytotoxicity of **1** was concentration-dependent in both cell lines, it was more pronounced at shorter times in MDA-MB-231 than in MCF-7.

**Fig. 3:**
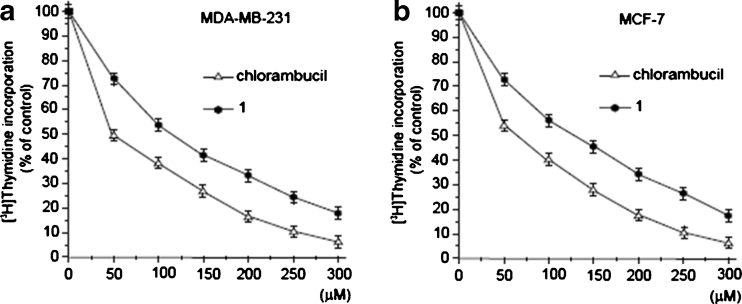
Cytotoxic effects of compound **1** and chlorambucil on the cultured breast cancer cell lines MDA-MB-231 (**a**) and MCF-7 (**b**) as measured by inhibition of [^3^H]-thymidine incorporation into DNA. Mean values ± SD of three independent experiments performed in duplicate are presented

The above data was corroborated by a cell proliferation assay. The profiles of DNA synthesis were found to be similar in MCF-7 and MDA-MB-231 (Fig. 3). The concentration of **1** required to inhibit [^3^H]-thymidine incorporation into DNA by 50 % (IC_50_) in MDA-MB-231 was found to be 123 ± 2 μM, suggesting a lower cytotoxic potency compared to chlorambucil (IC_50_ = 56 ± 2 μM). The concentrations of **1** and chlorambucil required for 50 % inhibition of [^3^H]-thymidine incorporation into DNA in breast cancer MCF-7 cells (IC_50_) were 124 ± 2 μM and 65 ± 2 μM, respectively.

### Computational studies

**Fig. 4:**
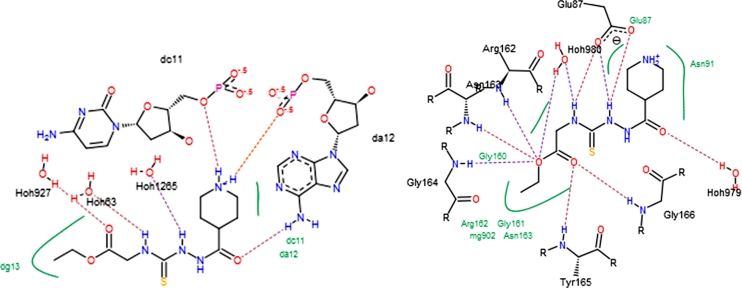
Interactions between **1** and the residues of the DNA-(*left*) and ATP-(*right*) binding sites

**Fig. 5:**
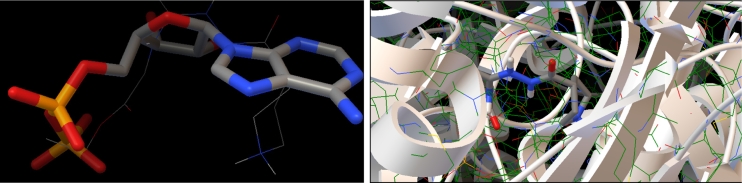
Docking results with ATP-binding domain of hTopoIIα (PDB: 1zxm).* Left* Superposition of the native ligand AMPPNP (rendered as* tubes*), and the best conformation of **1**.* Right* Binding mode of **1**

To examine the mode of inhibitory action of 4-ethoxycarbonylmethyl-1-(piperidin-4-ylcarbonyl)-thiosemicarbazide hydrochloride, **1**, docking simulations were performed with ATP-binding domain of hTopoIIα (PDB: 1ZXM) and DNA binding site of hTopoIIβ (PDB id: 3QX3) using the FlexX program, which has been shown to be reliable enough to carry out binding mode analysis of ligands in ATP binding pocket of hTopoIIα [31]. To validate the molecular docking protocol, AMPPNP and etoposide (bound ligands) were initially docked into the crystal structure of the enzyme. The docked ligands were found to have similar binding poses to the co-crystallized ligand, thus validating the adopted docking methodology. We then docked **1** into the DNA and ATP binding sites, respectively, and its interactions with the target enzyme were analyzed. In both cases, crystallographic water molecules were treated rotatable and displaceable. Their contribution to binding was found negligible in all cases. Free energies of binding of **1**, obtained from the Hyde scoring function [32, 33], are 9 kJ/mol in the DNA active site while it is −20 kJ/mol in the ATP binding site (vs −6 kJ/mol for AMPPNP, **2**, an analog of ATP crystallized with hTopoIIα). From this data we conclude that **1** has greater affinity toward the ATP binding site than to the DNA binding pocket of hTopoIIα and is an even more effective inhibitor than AMPPNP. Figures 4 and 5 show interactions of **1** in DNA and ATP active sites. As can be seen, compound **1** fits well into the ATP pocket, forming 11 hydrogen bonding interactions with water molecules, Gly164, Asn163, Arg162, Glu87, Gly166, Tyr165 and hydrophobic interactions with Gly160, Gly161, Asn91.

**Fig. 6:**
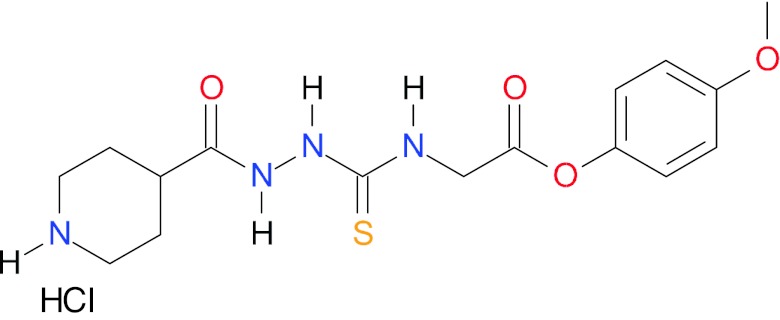
The chemical structure of 4-(4-methoxyphenyl)-1-(piperidin-4-ylcarbonyl)-thiosemicarbazide hydrochloride (**3**), which was found to be inactive as hTopo II inhibitor

To test the docking predictions, we repeated the docking simulations with the closely related structural analog of **1**, 4-(4-methoxyphenyl)-1-(piperidin-4-ylcarbonyl)-thiosemicarbazide hydrochloride **3** (Fig. 6), which was found to be inactive in our enzyme bioassays. Consistent with these results, the docking calculations correctly predict lack of stability of the active site—compound **3** complex (27 kJ/mol), confirming the reliability of the employed docking methodology.

**Fig. 7:**
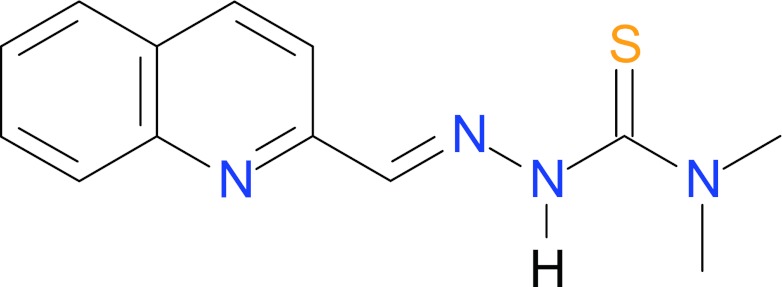
Chemical structure of thiosemicarbazone derivative, **4**—a recently identified hTopoIIα catalytic inhibitor [31]

Most recently, some meaningful results concerning the ligand recognition process in the ATP binging site were described by Ma and co-workers [16]. In this latter communication, the authors applied a combination of molecular docking approaches, DFT calculations, and CoMFA/CoMSIA methods to explore the possible binding modes of hTopo II inhibitors with the naphthoquinone core structure. Based on the best-scored conformations obtained from the FlexX docking simulation they generated the distribution of frontier molecular orbitals and found significant differences between potent and weak inhibitors in their HOMO distribution. The HOMO of the potent inhibitors covered almost the whole molecule while weak inhibitors had their HOMO outside the phenyl ring. It was thus proposed that distribution of HOMO significantly affects the binding of ligands to the ATP pocket. Following these findings, and using the same level of theory, we generated the HOMO orbitals for **1** and **3** and compared them with HOMO of the potent hTopoIIα catalytic inhibitor (which we refer to as **4**, Fig. 7) from the closely related class reported earlier in the literature [34]. Comparison of the orbitals leads to the conclusion that there is no similarity between HOMO distributions of the studied compounds and AMPPNP. A similar conclusion can be drawn from analysis of the LUMO distributions (MOs are given in Fig. S1 in the Supplementary Material). Thus it seems that the electronic distribution in the frontier orbitals is not related to inhibitory activity of studied compounds against Topo II—a conclusion in line with the lack of covalent bonding between inhibitor and active site residues.

## Conclusions

In this contribution we show compound **1** to be highly selective and potent inhibitor of human Topo II. From docking simulations, we conclude that its inhibitory action is connected with the ATP binding pocket although competitive inhibition assays are needed to confirm this hypothesis. These results will aid the rational design of novel thiosemicarbazide-based compounds that target the inhibition of Topo II and will provide insights into the discovery of novel anticancer agents.

## Electronic supplementary material

Below is the link to the electronic supplementary material.

ESM 1 (DOC 2533 kb)
