# Supplementary material for: Cytotoxic effect and molecular docking of 4-ethoxycarbonylmethyl-1-(piperidin-4-ylcarbonyl)-thiosemicarbazide—a novel topoisomerase II inhibitor
Source: J Mol Model. 2012 Nov 28;19(3):1319–24. doi: 10.1007/s00894-012-1679-6 (PMC3578725; doi:10.1007/s00894-012-1679-6)
Supplement: Supplementary file 1 — (DOC 2533 kb) [file 894_2012_1679_MOESM1_ESM.doc]

**Supplementary data**

**Cytotoxic effect and molecular docking of**

**4-etoxycarbonylmethyl-1-(piperidin-4-ylcarbonyl)-thiosemicarbazide, a novel topoisomerase II inhibitor**

Agata Siwek,^a,*^ Paweł Stączek,^b^ Monika Wujec,^a^ Krzysztof Bielawski,^c^ Anna Bielawska,^c^ Piotr Paneth^d^

*^a^Department of Organic Chemistry, Faculty of Pharmacy, Medical University, Chodźki 4a, 20-093 Lublin, Poland*

*^b^Department of Genetics of Microorganisms, University of Lodz, Banacha 12/16, 90-237 Lodz, Poland*

*^c^Department of Medicinal Chemistry and Drug Technology Medical University of Białystok, Kilinskiego 1, 15-089 Białystok, Poland*

*^d^Institute of Applied Radiation Chemistry, Faculty of Chemistry, Lodz University of Technology, Zeromskiego 116, 90-924 Lodz, Poland*

Contents

Title page S1

1. Molecular orbital maps of compounds **1-4**  S2-S3

1. Molecular orbital maps of compounds **1-4**

| 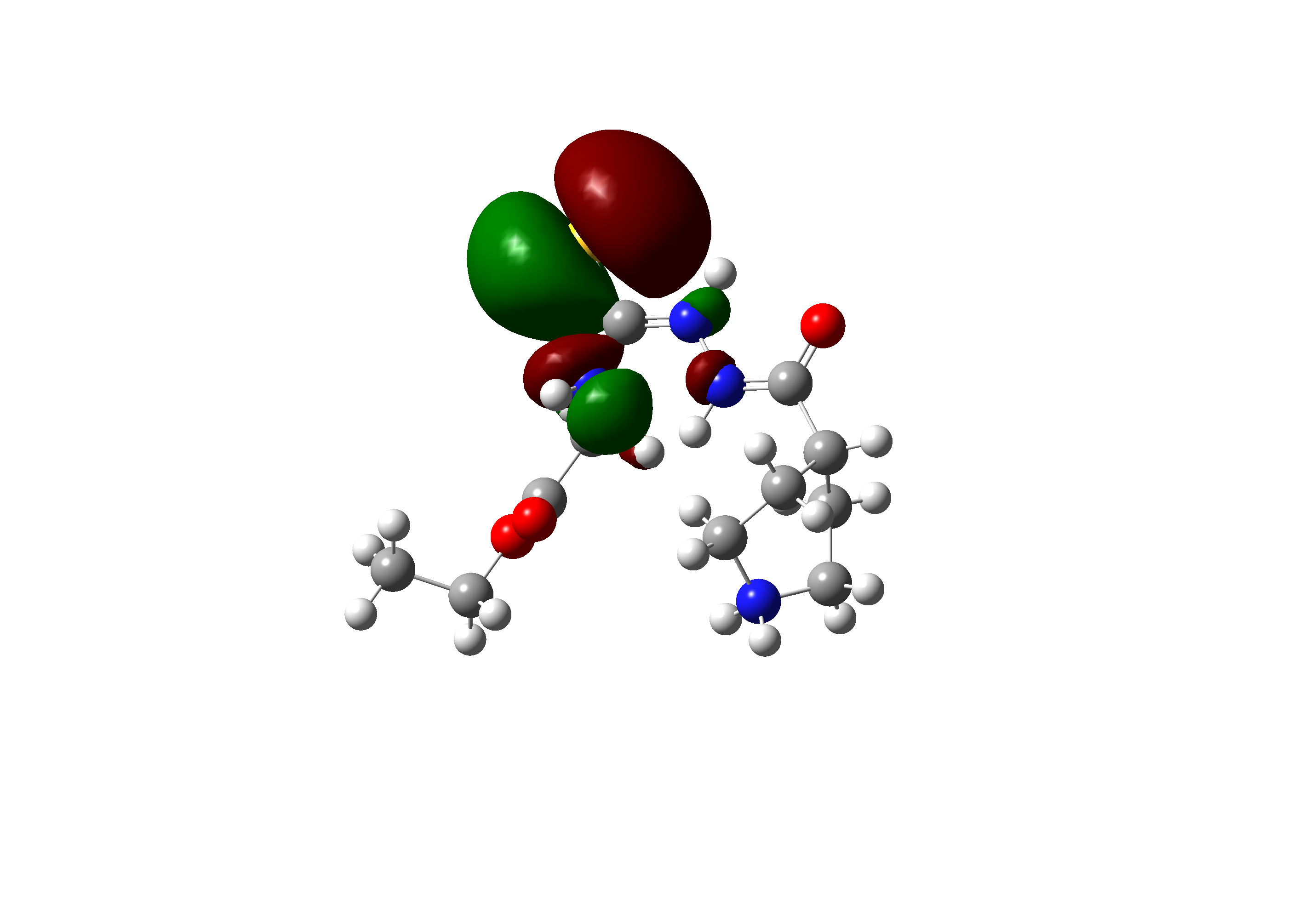 | 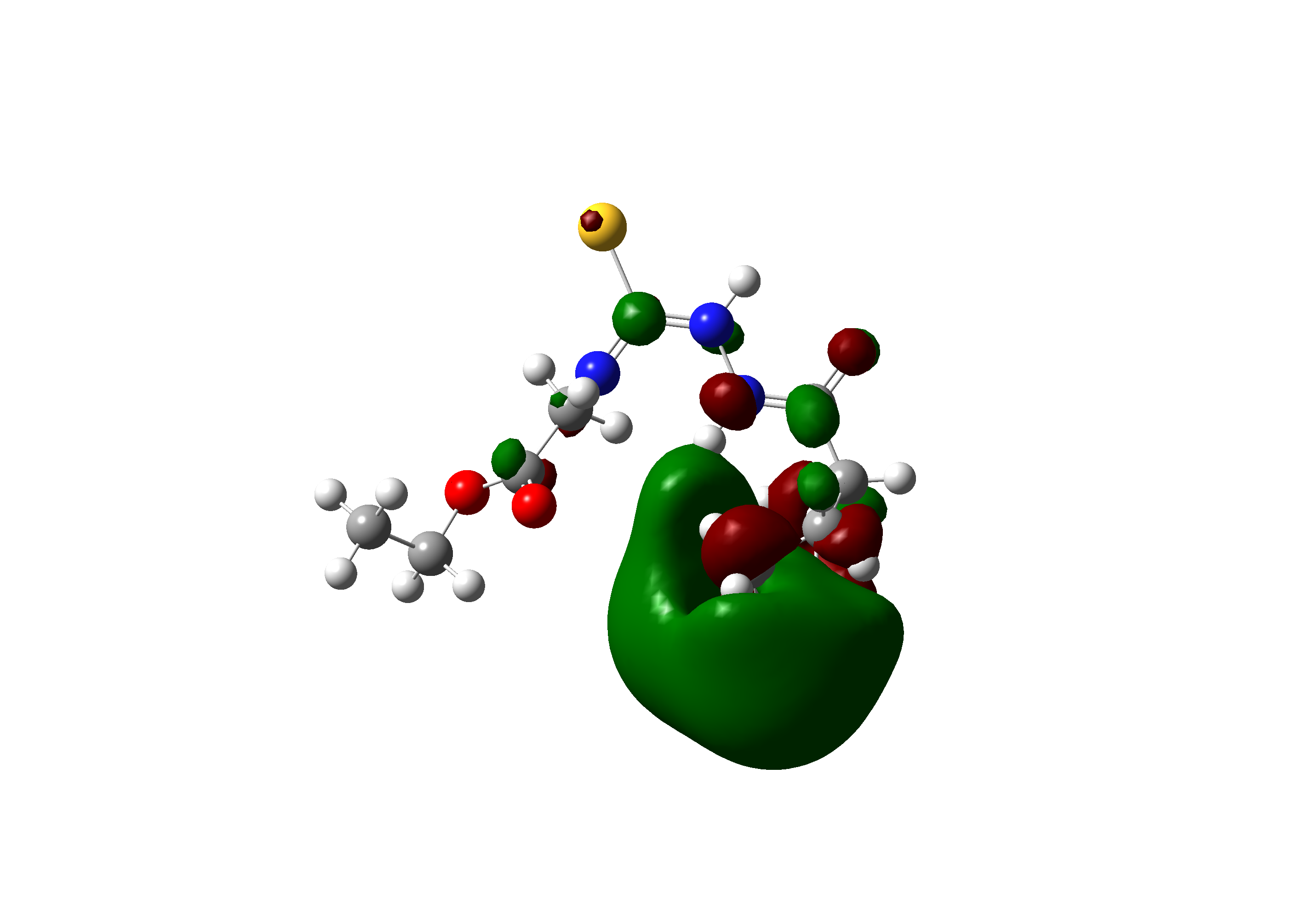 |
| --- | --- |
| **1** | |
| 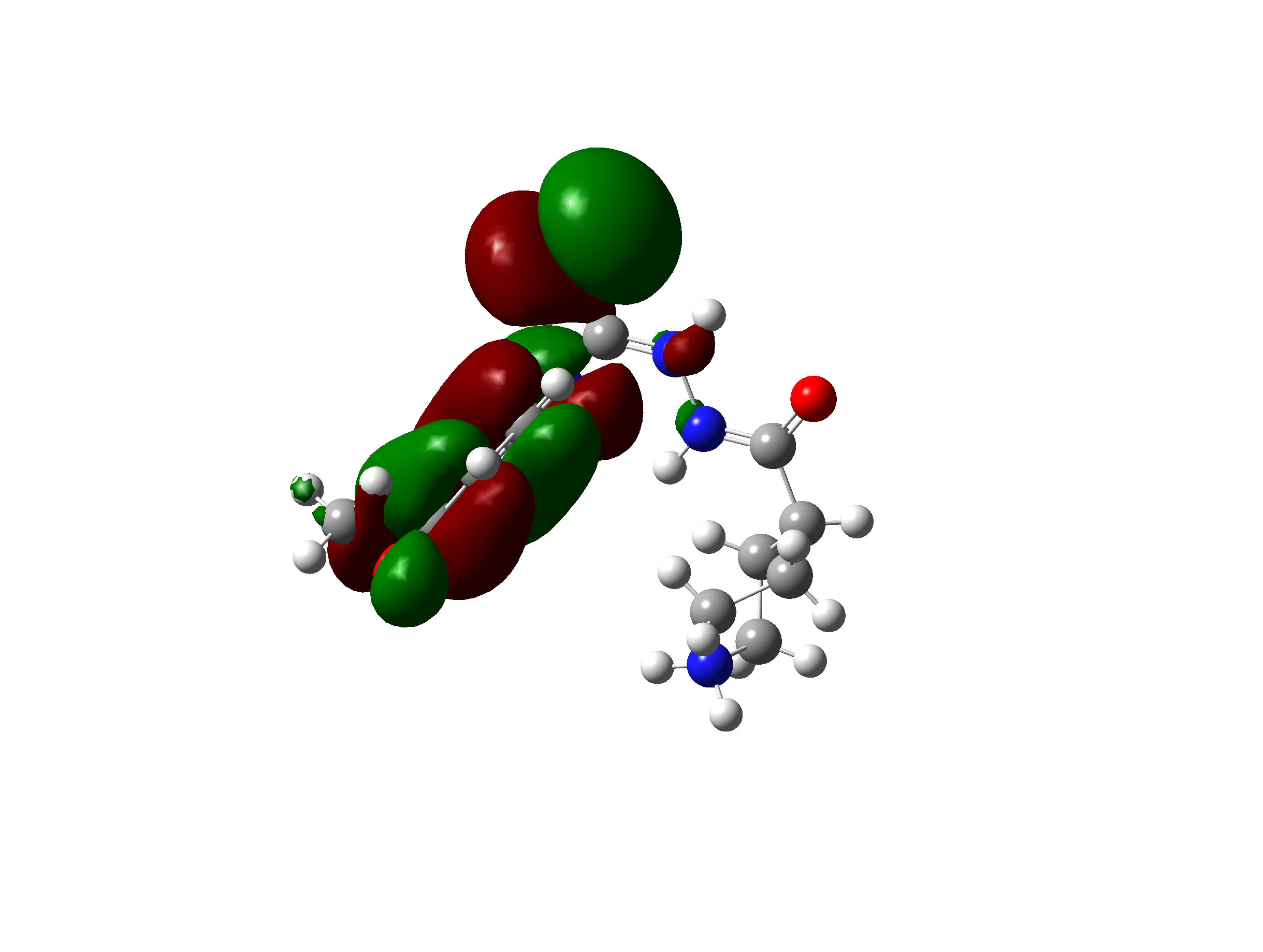 | 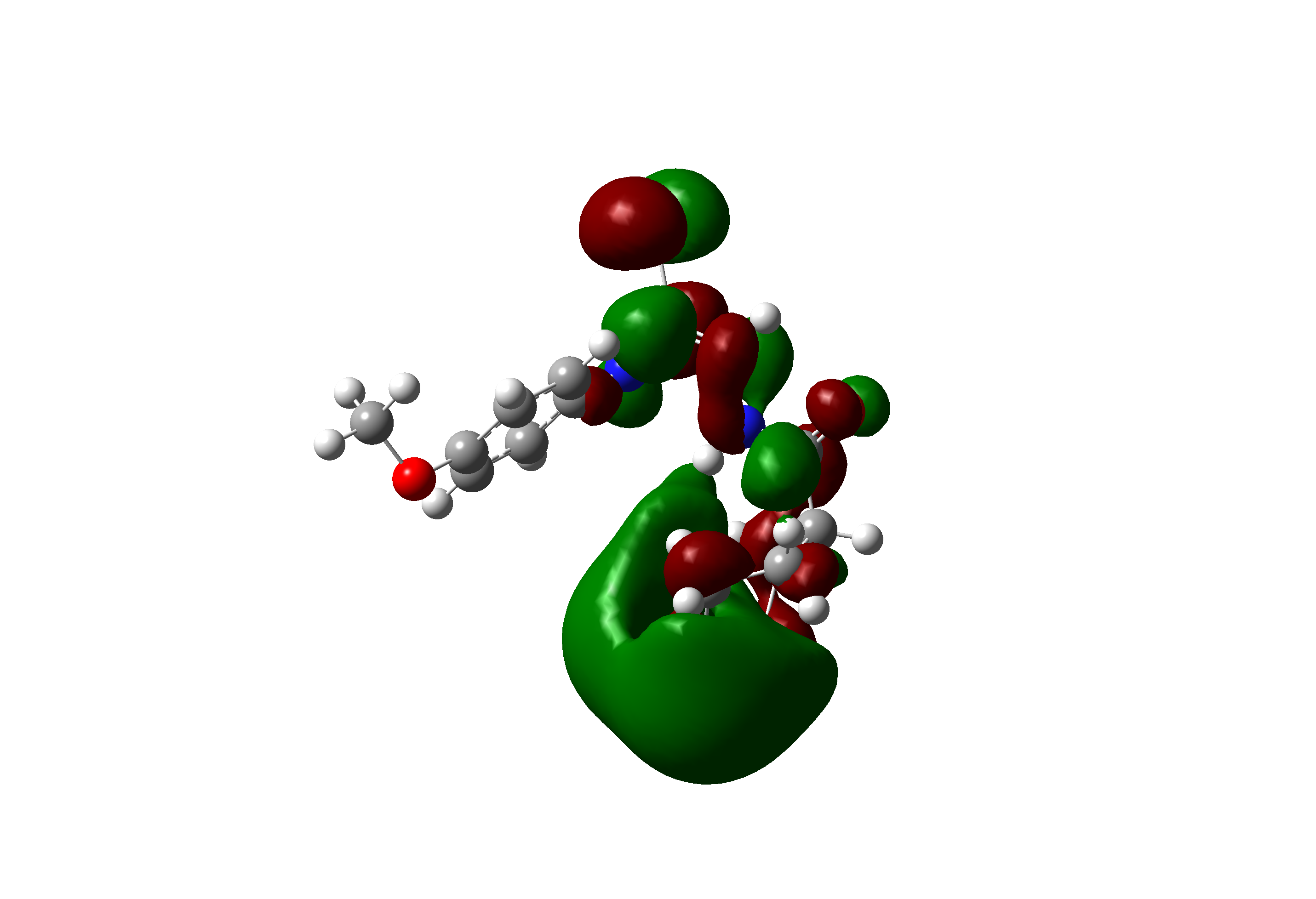 |
| **2** | |
| 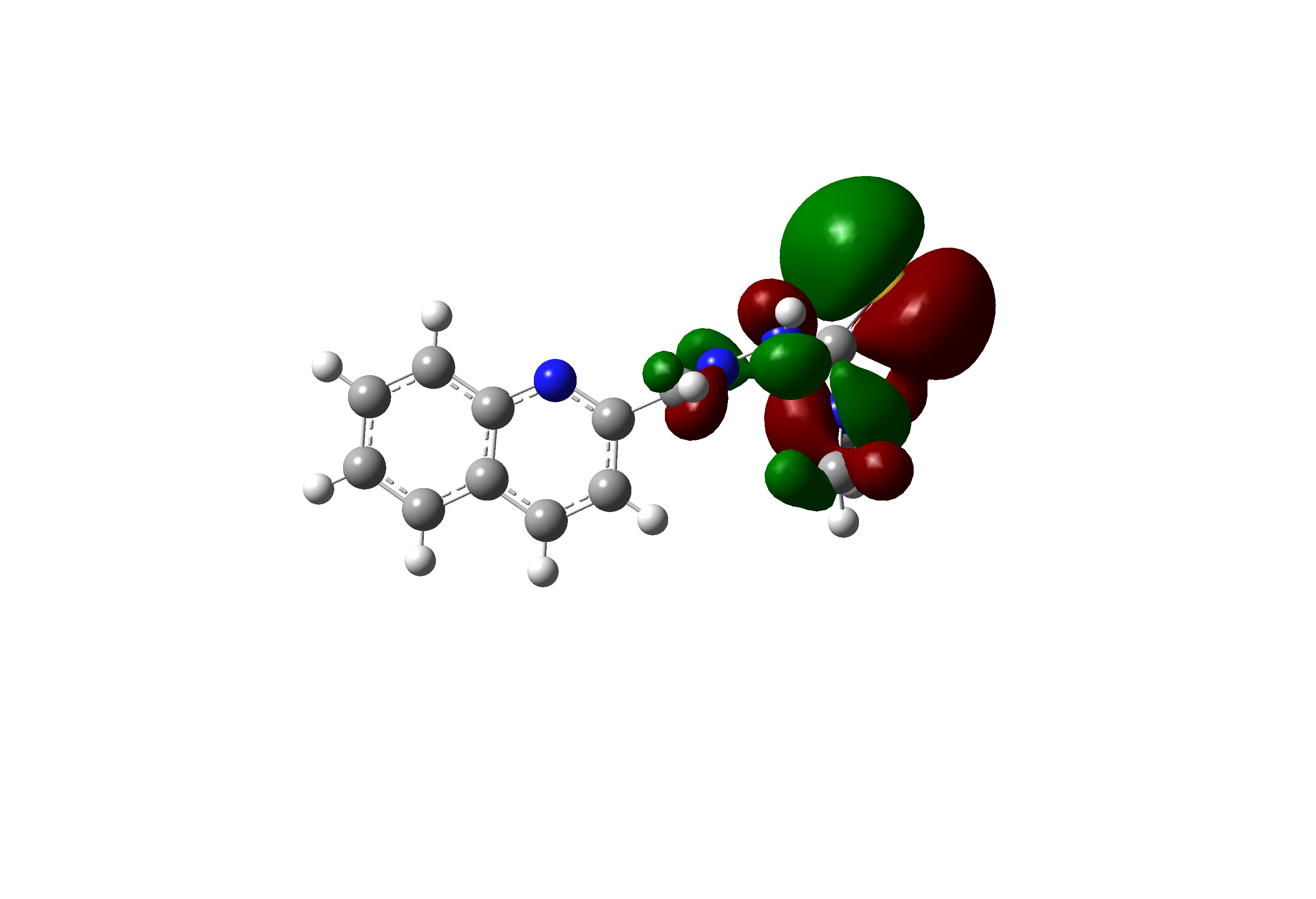 | 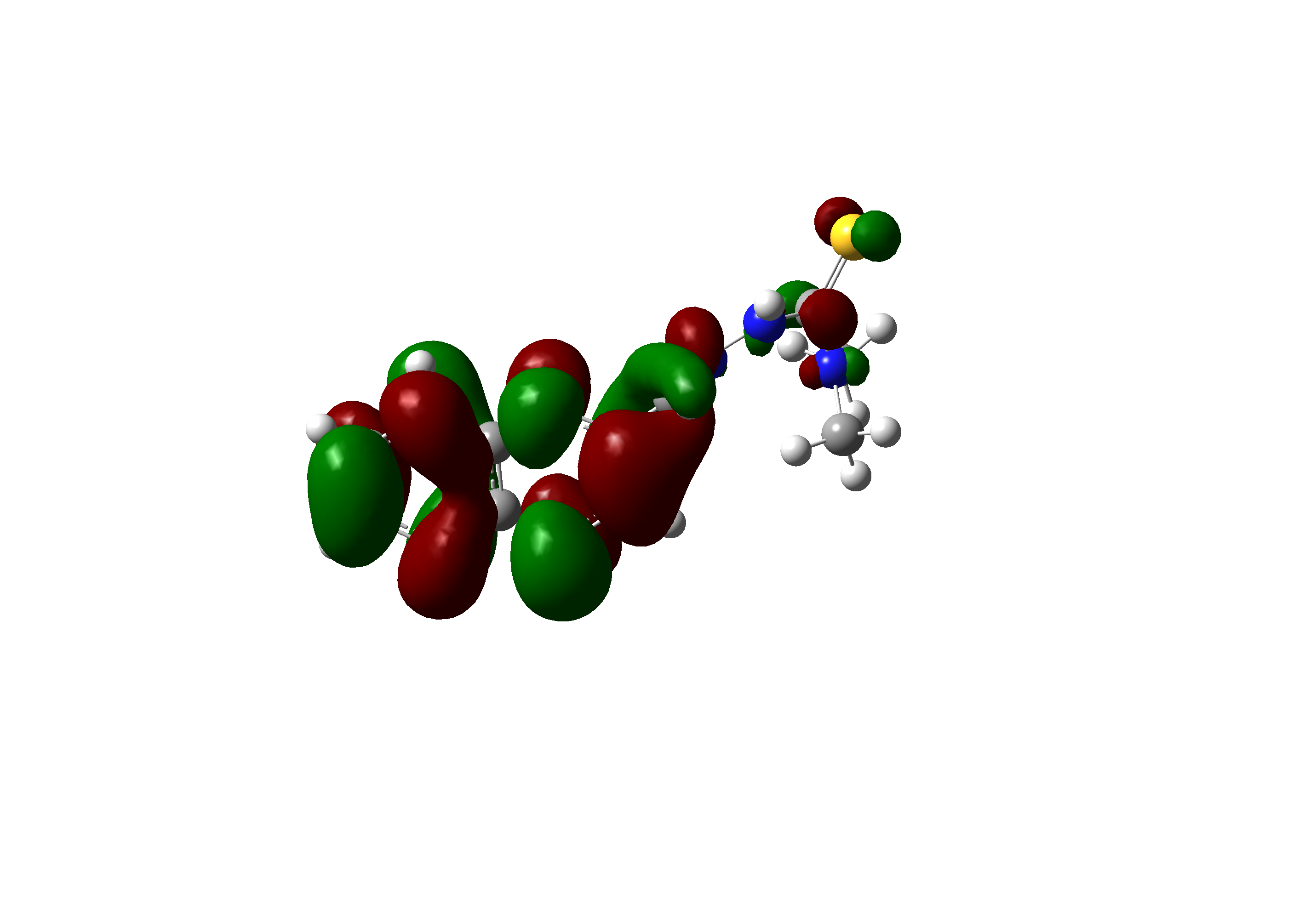 |
| **3** | |
| 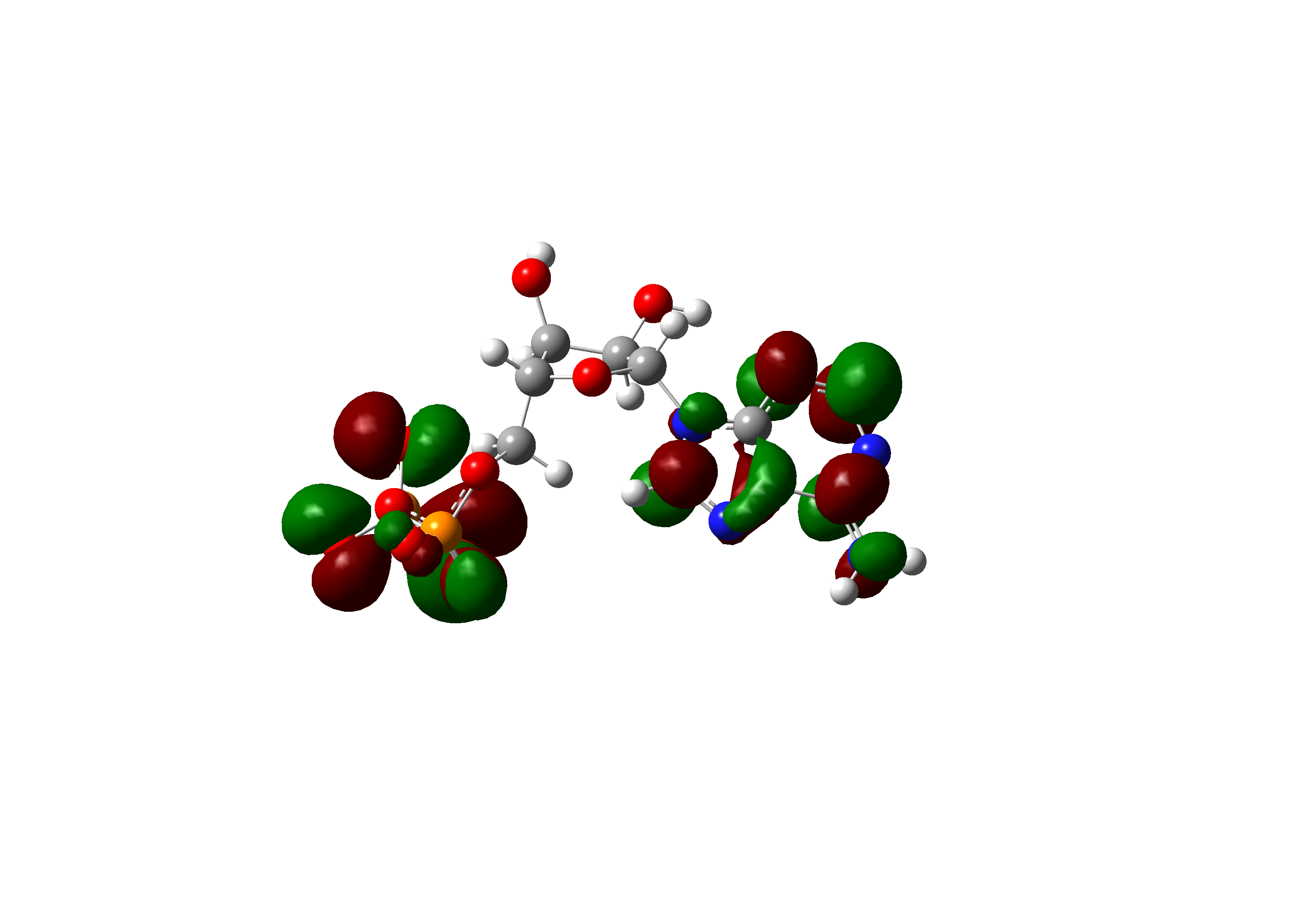 | 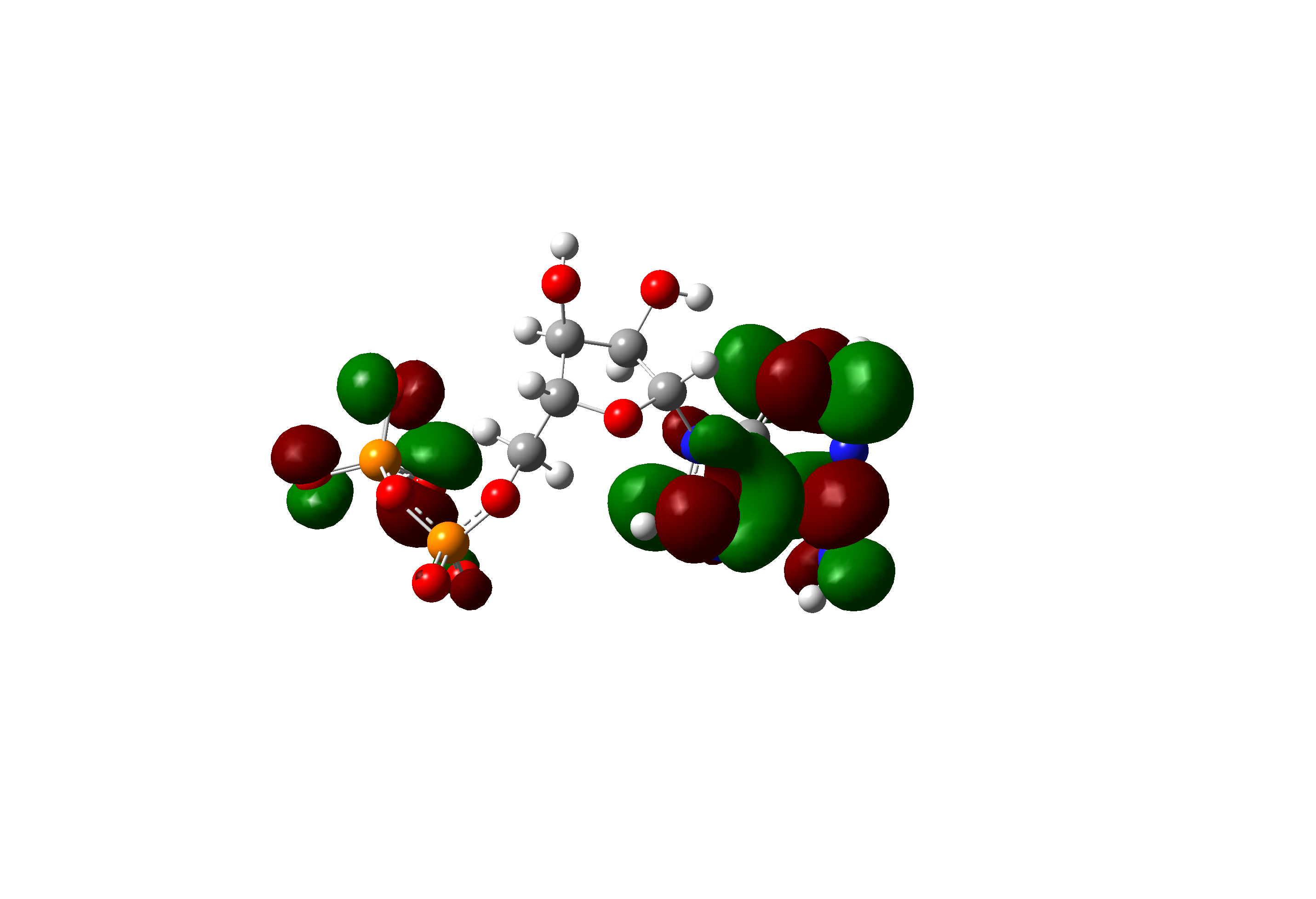 |
| **~~4~~** | |

**Fig. S1** HOMO (left) and LUMO (right) maps of **1-3** and AMPPNP **4**
